# Serum Interleukin-15 and its relationship with adiposity Indices before and after short-term endurance exercise

**DOI:** 10.12669/pjms.345.15516

**Published:** 2018

**Authors:** Mozaffer Rahim Hingorjo, Sitwat Zehra, Saima Saleem, Masood Anwar Qureshi

**Affiliations:** 1 Prof. Mozaffer Rahim Hingorjo, FCPS. Department of Physiology, Jinnah Medical & Dental College, Karachi, Pakistan; 2 Dr. Sitwat Zehra, PhD. Department of Physiology, Dr. A.Q. Khan Institute of Biotechnology & Genetic Engineering (KIBGE), Karachi, Pakistan; 3 Dr. Saima Saleem, PhD. Department of Physiology, Dr. A.Q. Khan Institute of Biotechnology & Genetic Engineering (KIBGE), Karachi, Pakistan; 4 Prof. Masood Anwar Qureshi, PhD. Department of Physiology, Dow University of Health Sciences, Karachi, Pakistan

**Keywords:** Interleukin-15, Myokines, Exercise, Adiposity

## Abstract

**Objective:**

The myokine interleukin-15 (IL-15) is capable of modifying the metabolism of both skeletal and adipose tissue. This study compares the change in serum levels of IL-15 in obese and non-obese after a single session of submaximal exercise.

**Methods:**

A cross-sectional study was carried out at Jinnah Medical and Dental College, Karachi, during Aug-Dec 2015, comprising of 133 medical students (aged 17-24 years). Cardiorespiratory fitness was evaluated by Queen’s College Step Test. Blood was obtained both before and just after exercise and serum levels of IL-15 determined by enzyme-linked immunosorbent assay.

**Results:**

Mean serum level of IL-15 was 3.64±1.59 pg/mL. Higher levels of IL-15 were seen in lean subjects compared to overweight/obese, both before and after three minutes of exercise (all P_trend_<.001). The percent increase in IL-15 upon exercise was 12.7% higher in lean. Significant negative association was seen between interleukin-15 and adiposity, especially visceral fat (*r* = –.288, *p*=.001).

**Conclusion:**

Interleukin-15 correlates negatively with adiposity indices, especially visceral fat. With the proven benefit of IL-15 in terms of adipose tissue stores and skeletal muscle mitochondrial biogenesis, endurance exercises, even of short duration, may possess therapeutic potential towards producing a healthier body.

## INTRODUCTION

Interleukin-15 is a myokine released from exercising muscles. First discovered in 1994 as an immune modulating agent,^1^ it is considered anabolic for proteins, enhancing myogenesis and providing protection against protein degradation.^2^ The release of IL-15 upon exercise also increases glucose uptake by translocating GLUT4 to the plasma membrane.^3^ Due to its anabolic and muscle building effects, it has also been called as the ‘Arnold cytokine’ after the body builder Arnold Schwartzenneger.^4^ The level of IL-15 depends upon the type, intensity and duration of exercise. Higher levels of IL-15 have been observed after both resistance and endurance training. In some studies, however, no change in plasma level of IL-15 was observed after acute exercise lasting 3 hours or less.^5^

Interleukin-15 apart from having anabolic activity, is also considered to reduce the total fat mass by increasing lipolysis and preventing the deposition of fat. A reduction in body weight and total fat mass was demonstrated when IL-15/soluble IL-15 receptor-alpha were given to obese mice on high fat diet.^6^ When given to obese leptin deficient mice, IL-15 was similarly observed to decrease the amount of white adipose tissue.^7^ Both the protein sparing and lipid oxidizing effects of IL-15 are thought to be mediated by peroxisome proliferator-activated receptor-delta (PPAR-δ), a mitochondrial activator.^8^ The regulation of adipose tissue mass by skeletal muscle release of IL-15 was studied by Nielsen et al., who found levels of IL-15 in plasma to have negative relationship with adiposity indices.^9^ This may be one way by which exercise prevents the development of central obesity and the disorders associated with it. The crosstalk of muscle with fat via IL-15 was also observed by Yang et al who found a higher plasma level of IL-15 in rats that underwent treadmill training along with an increase in the number of receptors for IL-15 on adipocytes.^10^

The association of IL-15 with different aspects of exercise such as type, intensity and duration, have not been completely explored. Furthermore, the release of IL-15 in response to few minutes of exercise has not been demonstrated. This study was conducted to measure the IL-15 response to submaximal endurance exercise lasting three minutes in obese and non-obese subjects.

## METHODS

This was a cross-sectional study with 133 subjects aged 17-24 years, selected from Jinnah Medical & Dental College, Karachi, during Aug-Dec 2015. A Physical Activity Readiness Questionnaire was filled by each to exclude any medical condition preventing exercise. Written informed consent was taken from each and the study was approved by the Research and Ethical Committee of Jinnah Medical and Dental College.

Weight was recorded digitally to the nearest 0.1kg while height was recorded to the nearest 0.1cm using seca 217 stadiometer. Body Mass Index (BMI) was calculated as the ratio of weight (kg) and height (m^2^) and subjects categorized into two groups according to Asian cutoff value: 11

***Group A:*** BMI <23.0 kg/m^2^

***Group B:*** BMI ≥23.0 kg/m^2^

Waist Circumference (WC) was measured while standing, midway between rib cage and iliac crest and Neck Circumference (NC) was measured just below the cricoid cartilage, both to the nearest 0.1cm. Bioelectric Impedance Analysis (BIA) was used to record percent Body Fat (%BF) and Visceral Fat (VF) (Omron HBF 510 Body Composition Monitor).

Cardiorespiratory fitness was measured by Queen’s College Step Test. The subjects were instructed not to do heavy physical work a day before and not to take food, caffeine, or smoke two hours before exercise. After noting resting pulse, the subjects were instructed to step up and down on a wooden platform (16.25 inch) in rhythm with metronome beats at a set rate for three minutes. After this the recovery pulse rate was counted to determine the subject’s VO_2_ max and read on an age-adjusted rating scale.^12^

Venous blood samples were obtained at rest and immediately post-exercise. Serum was isolated and stored at –40°C until analysis. Serum levels of IL-15 were detected using highly sensitive sandwich ELISA with the Human IL-15 ELISA kit (MBS705189) having sensitivity to detect lowest level of IL-15 = 0.78 pg/mL. The Intra- and inter-assay coefficients of variation were <8% and <10%, respectively.

Descriptive statistics (means ± SD) were used to evaluate the characteristics of each participant. Independent sample t-test was conducted to test differences in anthropometric and other variables between non-obese and obese groups. Pearson’s correlation coefficient was performed to determine the association of adiposity parameters with baseline serum IL-15 levels. The significance level was taken at p<.05. Statistical analyses was done using SPSS statistics (version 21.0).

## RESULTS

This study compared the change in serum IL-15 level after a single session of submaximal exercise in obese and non-obese individuals. Subject characteristics were studied separately by dividing them into group A (BMI < 23 kg/m^2^) and group B (BMI ≥ 23 kg/m^2^) Table-I. The mean age of participants was 19.37 ± 0.63 years and was not significantly different between the two groups. Forty four percent subjects were overweight/obese (group B) with a mean BMI of 27.25 ± 3.91 kg/m^2^. All adiposity indices including BMI, WC, WHtR, NC, %BF, and VF were significantly greater in group B (*p* < .001). The baseline IL-15 level (pg/mL) was 3.65 ± 1.59 (range 0.78–7.08) and increased significantly to 8.63 ± 3.92 (range 2.16–24.68). Gender variations in IL-15 were insignificant, however, IL-15 levels in lean subjects (Group A) were substantially higher compared to heavier subjects (Group B), both at baseline before the exercise (*p* = .001) and those taken after exercise (*p* < .001). Interleukin-15 levels were significantly higher in physically active subjects as compared to sedentary during resting state (p = .037) and the difference became highly significant immediately after exercise (p = .002).

**Table-I:** Comparison of characteristics between subjects having BMI < 23 kg/m^2^ and ≥ 23 kg/m^2^.

	Group A^a^	Group B^b^	p value
n (male/female)	75 (24 / 51)	58 (29 / 29)	
Age, years	19.4 (19.1-19.6)	19.4 (19.1-19.6)	.950
***Body Composition***			
Body Mass Index, kg/m^2^	19.6 (19.2-20.1)	27.7 (26.6-28.8)	< .001***
Waist Circumference, cm	74.8 (73.0-76.5)	94.1 (90.5-97.7)	< .001***
Neck Circumference, cm	32.3 (31.5-33.0)	36.3 (35.4-37.3)	< .001***
Waist-Height Ratio	0.46 (0.45-0.47)	0.57 (0.55-0.59)	< .001***
Percent Body Fat, %	22.9 (20.8-25.0)	35.6 (32.9-38.3)	< .001***
Visceral Fat Level	2.6 (2.3-2.9)	7.1 (6.3-7.7)	< .001***
***Interleukin-15, pg /mL***			
Before exercise	4.0 (3.7-4.4)	3.1 (2.8-3.5)	.001**
After exercise	9.8 (8.8-10.7)	7.2 (6.4-8.0)	<.001***

***Abbreviations:*** CI, confidence interval; SD, standard deviation. ***Note:*** Independent sample t test was used to compare means between groups A & B.

** p < .001, very significant,

*** p < .001, extremely significant.

a Group A = BMI < 23 kg/m^2^,

b Group B = BMI ≥ 23 kg/m^2^

**Fig.1:**
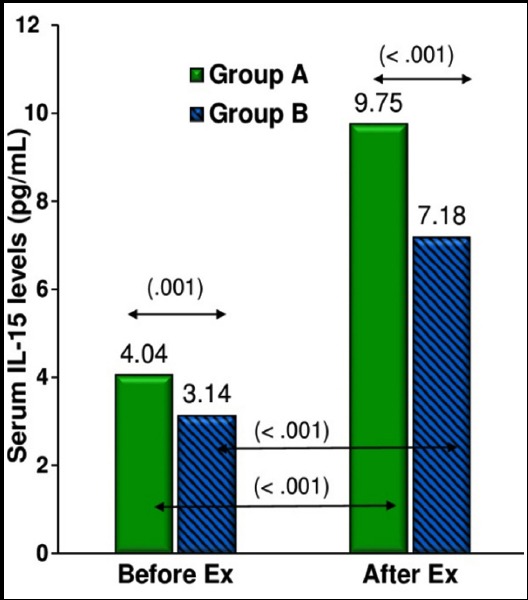
Comparison of serum IL-15 levels in subjects with BMI < 23 kg/m^2^ and BMI ≥ 23 kg/m^2^, before and after exercise. Group A, BMI < 23 kg/m^2^; Group B, BMI ≥ 23 kg/m^2^. *p* < .01, very significant; *p* < .001, extremely significant. ***Abbreviations:*** Ex, exercise; IL-15, Interleukin-15; BMI, body mass index

Fig.1 gives a composite picture of levels of IL-15 before and after exercise in lean and overweight/obese subjects. In response to exercise, serum levels of IL-15 significantly increased changing from 4.04 ± 1.60 to 9.75 ± 4.23 pg/mL (p < .001) in Group A and from 3.14 ± 1.44 to 7.18 ± 2.93 pg/mL (p < .001) in Group B.

**Fig.2:**
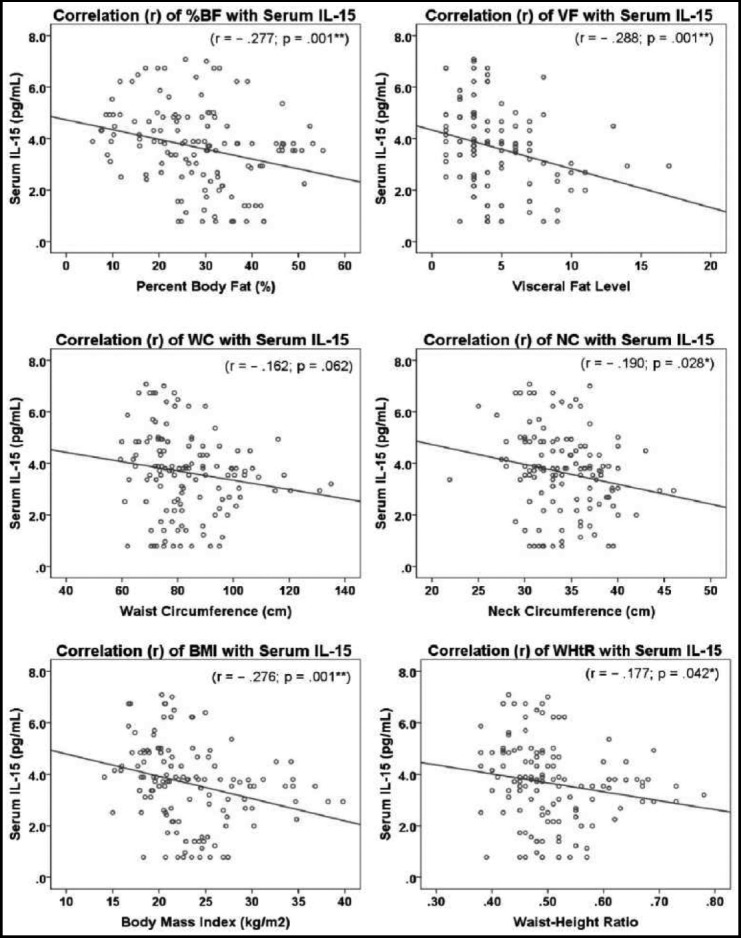
Scatter plot showing correlation (*r*) between pre-exercise serum interleukin-15 levels (IL-15) (pg/ml) and adiposity indices including Body Fat (BF) (%), Visceral Fat Level (VF), Waist Circumference (WC) (cm), Neck Circumference (NC) (cm), Body Mass Index (BMI) (kg/m^2^) and Waist-Height Ratio (WHtR) in study participants. p < .05 termed significant.

Interleukin-15 was observed to have significantly negative correlation with almost all the adiposity parameters. The highest correlation was seen with VF (*r* = – .288, *p* = .001), %BF (*r* = – .277, *p* = .001) and BMI (*r* = – .276, *p* = .001) (Fig.2).

## DISCUSSION

In this study, we investigated the association of IL-15 with body composition parameters and the response of IL-15 upon brief endurance exercise. Serum levels of IL-15 were significantly higher in lean as compared to overweight/obese subjects. Upon submaximal aerobic exercise of three minute duration, the serum levels of IL-15 significantly increased from baseline in both non-obese and obese subgroups but the increase was more prominent in the lean group.

The higher levels of IL-15 in lean individuals seem to represent a protective mechanism of this myokine towards adipose tissue accumulation. This relationship between IL-15 and adipose tissue in the context of exercise is explored in Table-II. Interleukin-15 has been suggested to work as a circulating myokine that prevents buildup of fat stores by enhancing energy expenditure. High circulating levels of IL-15 has been shown to cause a substantial decrease in body fat and prevent accumulation of adipose deposits resulting from ingestion of a high fat diet.^6^,^13^

**Table-II:** Literature Review of the Relationship Between Interleukin-15, Adipose Tissue and Skeletal Muscle.

Authors (year published)	Methodology	Outcome measures (results)	Comments
Nielsen *et al.* (2008)^9^	199 subjects divided into 4 groups based on obesity and T2DM	Plasma IL-15 was negatively associated with total fat mass (*p* < .05), trunk fat mass (*p* < .01), & % fat mass (*p* < .05) in multiple regression analysis adjusting for age, sex, fitness level, and smoking.	IL-15 may contribute to regulation of trunk fat mass.
Barra *et al.* (2010)^17^	Animal study to observe overexpression of IL-15 (IL-15tg), lack of IL-15 (IL-15^-/-^) upon adipose tissue	Overexpression of IL-15 (IL-15tg) was linked to lean body weight; lack of IL-15 (IL-15^-/-^) resulted in significant increase in weight gain without altering appetite.	IL-15 is involved in regulation of adipose tissue mass
Tamura *et al.* (2011)^20^	13 PA subjects performed 30 min endurance exercise at 70% maximum HR. Serum IL-15 pre- & post-exercise	Serum IL-15 increased significantly at 10 min & returned to resting level in 3 hr. Serum CK, biomarker for muscle damage, increased maximally 3 h post-exercise.	In response to endurance exercise, IL-15 may be released immediately and not related to muscle damage
Rinnov *et al.* (2014)^5^	12 week endurance training; 3 hr ergometer cycling. Plasma IL-15 & muscle biopsies before & after intervention	Endurance training induced 40% rise in basal skeletal muscle IL-15 protein content (p < .01). Acute exercise for 3-hr did not change muscle IL-15 or plasma IL-15 levels significantly.	IL-15 may take part in adaptation of skeletal muscle in response to endurance training.
Perez-Lopez *et al.* (2018a)^19^	14 subjects performed resistance exercise. Plasma IL-15 & muscle biopsies before & after exercise.	Serum IL-15 increased 5.3 fold immediately post-exercise (*p* < .001). Skeletal muscle IL-15Rα mRNA increased 2 fold 4 hours after exercise (*p* < .001).	In response to resistance exercise IL-15/IL-15Rα signaling pathway is activated in skeletal muscle
Perez-Lopez *et al.* (2018b)^21^	276 subjects divided into 5 groups based on PA, body mass and T2DM	Serum IL-15 & IL-15Rα was decreased in PA subjects compared to sedentary (*p* < .05) and increased in obese with T2DM compared to obese without T2DM (*p* < .05).	Circulating IL-15 and IL-15Rα are reduced in lean and obese PA subjects. IL-15Rα may play role in glucose metabolism
This Study (2018)	133 subjects divided into 2 groups based on BMI. Physical activity & fitness levels assessed	Serum IL-15 was higher in lean subjects compared to obese (*p* < .01). Physically active had higher IL-15 compared to sedentary (*p* < .01). Post-exercise level of IL-15 was 12.7% higher in lean.	IL-15 correlates negatively with adiposity indices, especially visceral fat. Levels of IL-15 rise early after the start of exercise

***Abbreviations:*** BMI, body mass index; PA, physical activity; IL-15, Interleukin-15; T2DM, type 2 diabetes mellitus; HR, heart rate; CK, creatine kinase

The present study also showed significant negative correlation of body composition variables with serum IL-15 levels. The highest degree of correlation was seen with central adiposity parameter, VF (*p* = .001). Interleukin-15 is a cytokine that inhibits lipid deposition in cultured adipocytes and decreases adipose tissue deposition in laboratory rodents. In human subjects, negative correlations between circulating IL-15 levels and both total and abdominal fat have been demonstrated.^14^ Nielson et al observed a negative correlation of plasma IL-15 levels and total fat mass (*p* < .001), BMI (*p* < .001) and trunk fat mass (*p* < .01).^15^ Animal experiments with knockout mice having deletion of *IL15* (IL-15 KO mice) demonstrated greater quantity of body fat than controls. On the other hand, transgenic mice that were engineered to secrete higher levels of IL-15 were found to have less fat and were resistant to weight gain in response to high fat diet.^16^ This may be due to the effect of IL-15 to increase adipose mitochondrial activity resulting in less fat accumulation and a lean stature.^8^,^17^

In our study, the subjects performed endurance type exercise for three minutes to a submaximal level after which more than 2-fold increase in serum levels of IL-15 was observed as compared to baseline (*p* < .001). Furthermore, the rise in serum IL-15 level was 12.6% more in lean subjects. Perez-Lopez et al has demonstrated a 5.3-fold increase in serum IL-15 levels in samples taken immediately after a session of resistance exercise.^18^ Tamura et al, observed a similar significant increase in IL-15 serum levels after 30 minutes of treadmill exercise performed at 70% of maximal heart rate. The rise was maximum 10 minutes after the end of endurance exercise, however, it was not sustained. The authors therefore speculated that IL-15 may have begun to rise immediately after or during the endurance exercise.^19^ This is supported by our study where we have observed a significant rise in serum IL-15 levels after only 3 minutes of endurance exercise.

We observed levels of IL-15 to be significantly higher in physically active subjects as compared to sedentary. This is in agreement with previous studies that demonstrate significant differences in IL-15 levels among sedentary individuals and those performing regular exercise, both resistant and endurance types. Regular endurance training has been shown to induce metabolic and oxidative adaptations in skeletal muscles including increase in basal levels of IL-15 protein in skeletal muscle by up to 40%.^5^ Animal studies have also demonstrated elevation in IL-15 levels in both skeletal muscle and serum after endurance as well as resistance training. This was associated with reduction of body weight and improvement in glucose sensitivity.^20^,^21^ Pérez-López et al., however, demonstrated lower levels of IL-15 and IL-15Rα in physically active subjects, both lean and obese.^22^ Quinn et al., identified IL-15 as an important regulator of mitochondrial oxidative enzymes inducing oxidative changes in skeletal muscle phenotype in response to endurance training.^23^

The beneficial effects of endurance exercise is related to effective mobilization and utilization of energy resources resulting in improved performance. This involves stimulation of key elements of oxidative metabolism such as PPARδ, sirtuin 1 and peroxisome proliferator-activated receptor-gamma coactivator (PGC)-1alpha. The end result is reduction in adiposity and insulin resistance while boosting up endurance performance. The role of IL-15 and other such anti-inflammatory ‘exerkines’ is still to be established.^9^ The ability of IL-15 to prevent or reduce adipose tissue deposits while promoting pro-oxidative changes in skeletal muscle makes it a potential pharmacological tool to be used as an exercise mimetic.

## CONCLUSION

Even though a pathophysiologic role cannot be inferred from the design of present study, the relationship between circulating IL-15 and several adiposity indices point to a participation of this myokine in overall physical fitness. Moreover, the early release of IL-15 upon performing physical activity, makes step-test a simple yet effective tool capable of inducing exercise related metabolic changes in the body. Additional investigations are required to observe the effect of training on the release of IL-15 in response to exercise of variable workload.

### Authors’ Contribution

**MRH, SZ & SS:** Conceived, designed and did statistical analysis & editing of manuscript.

**MRH, SZ, SS & MAQ:** Did data collection and manuscript writing, did review and final approval of manuscript.

## References

[1] Grabstein KH, Eisenman J, Shanebeck K, Rauch C, Srinivasan S, Fung V (1994). Cloning of a T cell growth factor that interacts with the beta chain of the interleukin-2 receptor. Science.

[2] O'Leary MF, Wallace GR, Bennett AJ, Tsintzas K, Jones SW (2017). IL-15 promotes human myogenesis and mitigates the detrimental effects of TNFαon myotube development. Scientific Reports.

[3] Krolopp JE, Thornton SM, Abbott MJ (2016). IL-15 activates the Jak3/STAT3 signaling pathway to mediate glucose uptake in skeletal muscle cells. Front Physiol.

[4] Teta J, Teta K, Pizzorno JE, Murray MT (2013). Therapeutic modalities. The exercise prescription. Textbook of Natural Medicine.

[5] Rinnov A, Yfanti C, Nielsen S, Akerstrom TC, Peijs L, Zankari A (2014). Endurance training enhances skeletal muscle interleukin-15 in human male subjects. Endocrine.

[6] Sun H, Ma Y, Gao M, Liu D (2016). IL-15/sIL-15Rαgene transfer induces weight loss and improves glucose homeostasis in obese mice. Gene Ther.

[7] Alvarez B, Carbo N, Lopez-Soriano J, Drivdahl RH, Busquets S, Lopez-Soriano FJ (2002). Effects of interleukin-15 (IL-15) on adipose tissue mass in rodent obesity models:evidence for direct IL-15 action on adipose tissue. Biochim Biophys Acta.

[8] Thornton SM, Krolopp JE, Abbott MJ (2016). IL-15 mediates mitochondrial activity through a PPARδ-dependent-PPARα-independent mechanism in skeletal muscle cells. PPAR Res.

[9] Nielsen AR, Hojman P, Erikstrup C, Fischer CP, Plomgaard P, Mounier R (2008). Association between interleukin-15 and obesity:interleukin-15 as a potential regulator of fat mass. J Clin Endocrinol Metab.

[10] Yang H, Chang J, Chen W, Zhao L, Qu B, Tang C (2013). Treadmill exercise promotes interleukin 15 expression in skeletal muscle and interleukin 15 receptor alpha expression in adipose tissue of high-fat diet rats. Endocrine.

[11] WHO Expert Consultation (2004). Appropriate body-mass index for Asian populations and its implications for policy and intervention strategies. Lancet.

[12] McArdle WD, Katch FI, Pechar GS, Jacobson L, Ruck S (1972). Reliability and interrelationships between maximal oxygen intake, physical work capacity and step-test scores in college women. Med Sci Sports.

[13] Duan Y, Li F, Wang W, Guo Q, Wen C, Li Y, Yin Y (2017). Interleukin-15 in obesity and metabolic dysfunction:current understanding and future perspectives. Obes Rev.

[14] Quinn LS, Anderson BG (2011). Interleukin-15, IL-15 receptor-alpha, and obesity:concordance of laboratory animal and human genetic studies. J Obes.

[15] Nielsen AR, Mounier R, Plomgaard P, Mortensen OH, Penkowa M, Speerschneider T (2007). Expression of interleukin-15 in human skeletal muscle effect of exercise and muscle fiber type composition. J Physiol.

[16] Barra NG, Reid S, MacKenzie R, Werstuck G, Trigatti BL, Richards C (2010). Interleukin-15 contributes to the regulation of murine adipose tissue and human adipocytes. Obesity (Silver Spring).

[17] Barra NG, Palanivel R, Denou E, Chew MV, Gillgrass A, Walker TD (2014). Interleukin-15 modulates adipose tissue by altering mitochondrial mass and activity.

[18] Perez-Lopez A, McKendry J, Martin-Rincon M, Morales-Alamo D, Perez-Kohler B, Valades D (2018). Skeletal muscle IL-15/IL-15Rαand myofibrillar protein synthesis after resistance exercise. Scand J Med Sci Sports.

[19] Tamura Y, Watanabe K, Kantani T, Hayashi J, Ishida N, Kaneki M (2011). Upregulation of circulating IL-15 by treadmill running in healthy individuals:is IL-15 an endocrine mediator of the beneficial effects of endurance exercise?. Endocr J.

[20] Molanouri Shamsi M, Hassan ZM, Quinn LS, Gharakhanlou R, Baghersad L, Mahdavi M (2015). Time course of IL-15 expression after acute resistance exercise in trained rats:effect of diabetes and skeletal muscle phenotype. Endocrine.

[21] Kim HJ, Park JY, Oh SL, Kim YA, So B, Seong JK (2013). Effect of treadmill exercise on interleukin-15 expression and glucose tolerance in zucker diabetic Fatty rats. Diabetes Metab J.

[22] Pérez-López A, Valadés D, Vázquez Martánez C, de Cos Blanco AI, Bujan J, Garcáa-Honduvilla N (2018). Serum IL-15 and IL-15Rαlevels are decreased in lean and obese physically active humans. Scand J Med Sci Sports.

[23] Quinn LS, Anderson BG, Conner JD, Wolden-Hanson T (2013). IL-15 overexpression promotes endurance, oxidative energy metabolism, and muscle PPARδ, SIRT1, PGC-1α, and PGC-1βexpression in male mice. Endocrinology.

